# Evaluating the budget impact of Empagliflozin in managing heart failure with reduced ejection fraction: Proposing strategic policies for Malaysian public healthcare

**DOI:** 10.1371/journal.pone.0313131

**Published:** 2024-10-31

**Authors:** Vee Sim Yong, Sivaraj Raman, Chia How Yen, Mohd Shahri Bahari, Nur Amalina Zaimi, Houng Bang Liew

**Affiliations:** 1 Clinical Research Centre, (Hospital Queen Elizabeth II), Institute for Clinical Research, National Institute of Health, Ministry of Health Malaysia, Kota Kinabalu, Sabah, Malaysia; 2 Centre for Health Economics Research, Institute of Health Systems Research, National Institute of Health, Ministry of Health Malaysia, Kota Kinabalu, Sabah, Malaysia; 3 Cardiology Department, Hospital Queen Elizabeth II, Ministry of Health Malaysia, Kota Kinabalu, Sabah, Malaysia; Tehran University of Medical Sciences, ISLAMIC REPUBLIC OF IRAN

## Abstract

Sodium-glucose co-transporter-2 (SGLT2) inhibitors such as Empagliflozin, are increasingly recommended as part of guideline-directed medical therapy (GDMT) for heart failure with reduced ejection fraction (HFrEF) in many developed nations. This recommendation is based on robust clinical evidence showing that adding Empagliflozin to GDMT improves heart failure symptoms, clinical outcomes, functional status, and overall quality of life. In Malaysia, where healthcare is predominantly public and heavily subsidized, the introduction of new treatments can significantly impact costs, requiring detailed economic assessments. This study evaluates the budget impact of incorporating Empagliflozin into GDMT for HFrEF from the perspective of the public healthcare system. A five-year budget impact model was developed, integrating local data such as population, drug use, costs, clinical outcomes, and healthcare expenses. In the current scenario (GDMT alone), the projected five-year expenditure is MYR 6.12 billion (USD 3.92 billion). With Empagliflozin, the total cost rises by 0.71% to MYR 6.16 billion (USD 3.95 billion), driven by drug acquisition costs of MYR 160.12 million (USD 102.64 million) and adverse event costs of MYR 211,543 (USD 135,604). However, these costs are offset by savings from reduced HF hospitalizations, fewer cardiovascular deaths, and improved renal outcomes. Sensitivity analysis identified hospitalization costs, the price of Empagliflozin, and cardiovascular deaths in diabetic patients as key factors influencing the budget impact. Policymakers can improve the affordability of Empagliflozin through strategies like price negotiations, cost-sharing, and focusing on high-risk groups to optimize healthcare expenditure while ensuring effective treatment access.

## Introduction

Heart failure (HF) is a clinical syndrome of the terminal stages of most cardiovascular diseases. Globally, it affects 64 million people worldwide, imposing a substantial economic burden estimated at USD 346.2 billion [1]. A significant subset of these patients will progress to heart failure with reduced ejection fraction (HFrEF), characterised by a left ventricular ejection fraction (LVEF) of less than 40%. This stage is marked by high clinical deterioration, with nearly one-third of patients experiencing worsening symptoms within the first year [2]. In Malaysia, the incidence of HF approximately ranges from 3 to 20 cases per 1,000 population, with HFrEF forming 64.9% of these cases [3, 4]. The high morbidity and mortality associated with HFrEF are exacerbated by a prevalent incidence of diabetes mellitus (DM) and dyslipidemia, alongside an aging population [5, 6]. Such a burden translated into a projected direct cost of MYR 766.3 million (USD 481.9 million) in 2021 [7]. This rising burden underscores the need for more effective treatments and healthcare strategies to mitigate the impact of HFrEF on both patients and healthcare systems.

Pharmacotherapy remains the cornerstone of treatment for HFrEF, with a primary aim of prevention of recurrent hospitalizations due to worsening HF [8]. Treatment includes a combination of Guideline Directed Medical Therapy (GDMT) consisting of beta-blockers, angiotensin-converting enzyme inhibitors (ACEi), angiotensin receptor blockers (ARB), angiotensin receptor and neprilysin inhibitors (ARNi), and mineralocorticoid receptor antagonist (MRA). Recently, sodium-glucose co-transporter 2 (SGLT2) inhibitors, such as Empagliflozin and Dapagliflozin, have been incorporated as additions to the GDMT. This recommendation is supported by evidence demonstrating that this therapeutic combination not only alleviates symptoms but also improves clinical outcomes, functional capacity, and quality of life [9–11]. Initially approved for the treatment of DM, several randomised controlled trials have since shown that SGLT2 inhibitors also significantly reduce the risk of HF hospitalisations. Notably, this benefit has been observed in both diabetic and non-diabetic populations [10–12].

In Malaysia, the National Medicines Formulary regulates prescribing in public healthcare institutions. Both Empagliflozin and Dapagliflozin are listed in the formulary and are indicated to reduce the risk of cardiovascular death and hospitalization in HFrEF. The recent Malaysian Clinical Practice Guidelines Management of Heart Failure 2023 has also included SGLT2 inhibitors as one of the foundational HF medications [13]. However, despite their inclusion, ensuring equitable access to such treatments remains a challenge, largely due to affordability concerns. This issue is further compounded by the limited availability of local data on cost-effectiveness, which hampers resource planning and allocation. While the initial acquisition costs of adding SGLT2 inhibitors to GDMT for HFrEF may increase short-term expenditures, these costs could be offset by long-term reductions in the overall economic burden of HFrEF.

A recent study evidenced a modest increment in budget for the addition of Dapagliflozin in HFrEF management in Malaysia [14]. Nevertheless, an earlier local comparative analysis demonstrated that Empagliflozin may be a relatively more affordable treatment option [15]. Given the heavily subsidized nature of Malaysia’s public healthcare system, any expansion of Empagliflozin’s use still requires a comprehensive financial assessment [16]. To address these concerns, a budget impact analysis was conducted from the perspective of the Malaysian Ministry of Health. This analysis estimates the net financial implications of incorporating Empagliflozin into GDMT for HFrEF and identifies key factors influencing its affordability. The findings offer crucial insights for policymakers, aiding the development of strategies to optimize resource use while improving access to this potentially life-saving treatment.

## Methods

A budget impact analysis addresses the expected changes in healthcare expenditure after the adoption of a new treatment. This is achieved by first developing a robust computing framework that allows local input values to be applied to generate potential outputs. In this study, the budget impact model was designed following the recommendation from the International Society for Pharmacoeconomics and Outcomes Research (ISPOR) Task Force [17]. It consists of the eligible population, treatment options being explored, and all relevant expenditures.

### Analytical framework

**Fig 1:**
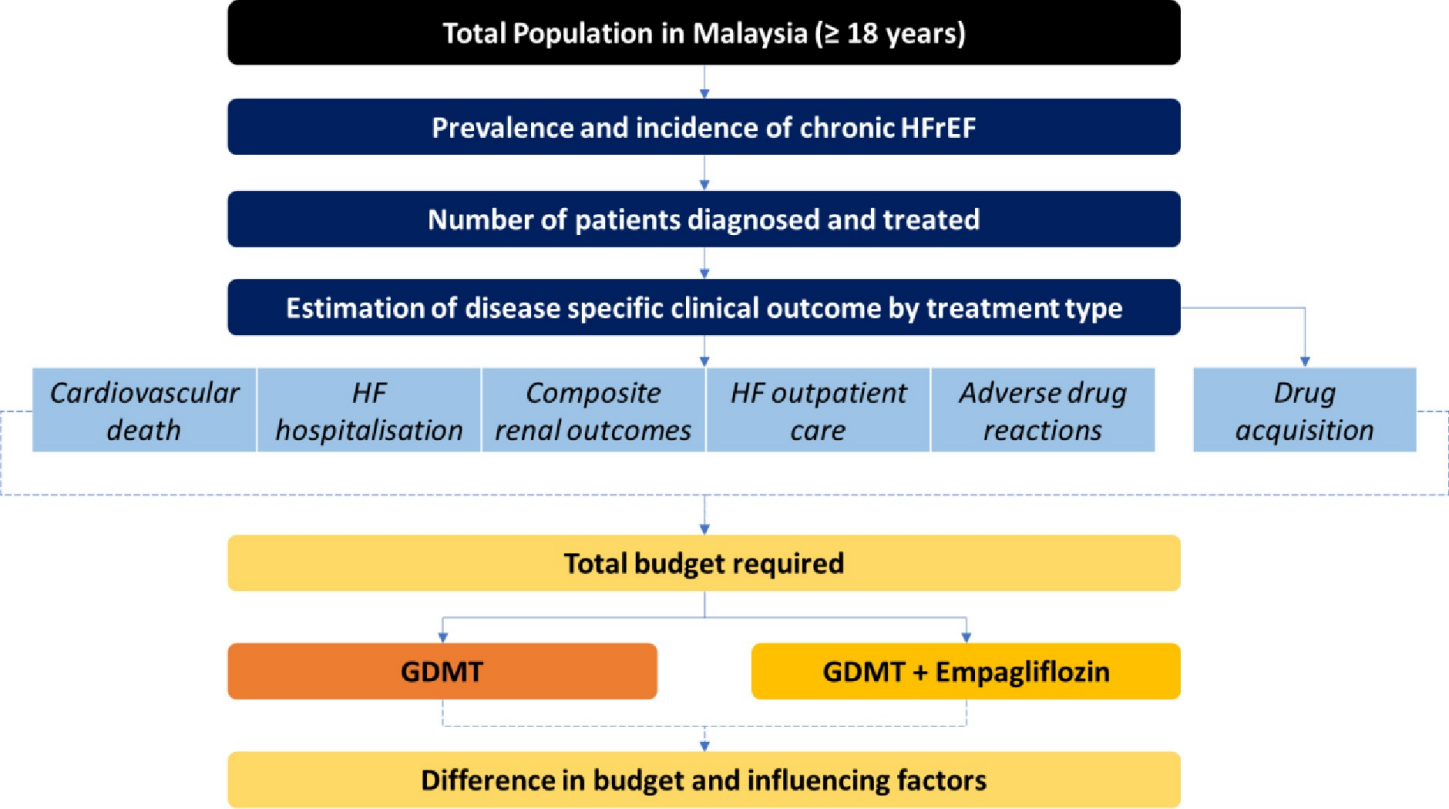
Budget impact model structure of current scenario (GDMT alone) and new scenario (GDMT + Empagliflozin).

The analytical framework for the budget impact analysis was adapted from a similar study conducted from a commercial payer perspective in the United States [18]. The model was constructed in Microsoft Excel. It explores the progression of patients from being diagnosed with HFrEF to being initiated with either GDMT or GDMT plus Empagliflozin, followed by their outcomes and survival (Fig 1). The financial impact consisted of the expenditures for drug acquisition and management of all relevant HFrEF events. As patients with and without DM have separate treatment and disease outcomes, both the patient sub-groups were simulated separately within the model.

The analysis was conducted from the Malaysian Ministry of Health’s perspective as public healthcare is predominantly funded by taxation and general revenues from the federal government. The time horizon was set annually and for a period of five years. This is following the budgeting process in the Ministry of Health and the recommendation from the ISPOR Task Force and Malaysian Pharmacoeconomics Guideline [17, 19]. Local input parameters, encompassing population data, drug utilisation and costs, clinical outcomes, and their corresponding costs, were incorporated wherever available. In instances where necessary, the insights of cardiologists and the management team were sought.

### Eligible population

The eligible population data were obtained by first estimating the total number of adults in Malaysia above 18 years of age [20]. Heart failure prevalence data of 0.72% was applied to this population to estimate the number of current patients with HF [21]. This was followed by applying an estimated diagnosis dropout of 20% and treatment compliance of 70%. The proportions were based on the opinions of cardiologists in tertiary care centers as no local values were available. Out of this hypothetical eligible HF population for treatment, 64.9% of them were assigned to have HFrEF. This was according to the findings from the Malaysian Heart Failure (MyHF) registry [4]. A total population approach was adopted as all Malaysian citizens can access public healthcare without any restrictions.

The eligible HFrEF population was further split into those with DM (58%) and those without (42%) [22]. This was because HFrEF with DM has a significantly higher risk of poorer clinical outcomes and mortality [12]. Simulation of two cohorts with and without DM ensures that disease-specific mortality and morbidity probabilities can be applied to generate more accurate clinical outcomes. In subsequent years, the dynamics of the patient population encompass a mix of newly diagnosed individuals and patients from the preceding year who remained alive in the current year. The population estimates are reported in the results section.

### Drug utilisation and cost

In the current scenario, eligible patients were assumed to receive optimal treatment with GDMT drugs. Following the recommendations outlined in the Malaysian Clinical Practice Guidelines Management of Heart Failure, this includes a combination of beta-blockers, ACEi, ARB or ARNi, and MRA [3]. The rate of utilisation of each drug was obtained from an internal patient registry in Hospital Queen Elizabeth II. This tertiary public hospital, located in the state of Sabah, serves as the state cardiology referral center. The rates were derived from the follow-up data of 130 HFrEF patients over one year from September 2020 to September 2021. For ease of estimation, drug utilisation rates were assumed to remain constant over time. These drug utilisation rates were applied to all eligible treated patients to generate total drug consumption.

For the projected new scenario, it was assumed that 10% of patients treated with GDMT would receive Empagliflozin in the first year. This was followed by a market share of 15% in the second year and 20% from the third to the fifth year. The market share was based on expert opinions and the feasibility of the introduction of a new agent into the local formulary. As Empagliflozin is contraindicated for patients with glomerular filtration rate (eGFR) lesser than 30 ml/min/1.73m^2^, it was assumed that such patient sub-group did not receive the GDMT plus Empagliflozin plus GDMT regimen [23]. The drug utilisation rates of GDMT were similar in the new scenario, as Empagliflozin serves only as an add-on rather than a replacement for any of the GDMT components.

Drug acquisition costs were calculated based on cost per tablet multiplied by the total number of tablets required annually for the management of HF. Drug wholesale acquisition costs were sourced from the Public Sector Medicines Wholesale Price List, made available by the Pharmacy Division, Ministry of Health Malaysia 2022. It is worth highlighting that drug prices in public healthcare are based on the currently available form (originator or generic) in the formulary. These prices may vary with the retail market price due to government price negotiations and tender contracts. Target drug doses were based on either the commonly prescribed doses or the maximum doses indicated for the management of chronic HF [3]. Patients were assumed to continue treatment until death, with no discontinuation and dose changes. These annual drug costs were later multiplied by the total number of eligible patients generated in the model in the respective year.

### Clinical outcomes and cost

The model focused on four primary clinical outcomes: cardiovascular-related deaths, HF hospitalisation, composite renal outcomes, and drug-related adverse events. The likelihood of these outcomes was contingent on the specific treatment scenarios. Due to the absence of local evidence, data on clinical outcomes were sourced from landmark trials and relevant literature (Table 1). Composite renal outcomes were integrated into the model, considering that the addition of Empagliflozin was observed to result in a slower progressive decline in renal function among HFrEF patients [10]. As for adverse events, the analysis only considered urinary tract infections and genital mycotic infections. This selection was based on findings from the EMPEROR-Reduced Trial, which indicated that only these two types of adverse events exhibited significant differences in their frequency between GDMT and GDMT plus Empagliflozin [24].

**Table 1:** Clinical outcome inputs^a^.

Input	Unit	Current Scenario (GDMT)	New Scenario (GDMT + Empagliflozin)
**Cardiovascular Death**	events/100 patient-year		
**HFrEF without DM**		7.3%	6.795%
**HFrEF with DM**		9.1%	8.42%
**HF Hospitalisation**	events/100 patient-year		
**HFrEF without DM**		12.35%	9.235%
**HFrEF with DM**		18.62%	12.36%
**Composite renal outcome**	events/100 patient-year		
**HFrEF without DM**		2.05%	0.955%
**HFrEF with DM**		4.17%	2.3%
**Adverse Events**	events/100 patient-year		
**Urinary Tract infection**		3.74%	4.13%
**Genital mycotic infection**		0.54%	1.38%

^*a*^*Anker et al* [12]

### Medical costs

The cost of HF hospitalisation was obtained from published local studies which utilised local registry data. This activity-based costing study identified the annual cost of HF hospitalisation, which included in-patient stays, medications, diagnostic tests, and procedures [25]. As there was no local data on cardiovascular death costs, the value was estimated based on the terminal care costs approach. Several studies illustrated that the healthcare costs for HF patients in the final months of life were nearly three times higher than their annual hospitalisation cost [26]. Consequently, the cost of cardiovascular death was estimated to be 2.36 times the local HF hospitalisation cost, reflecting the substantial utilisation of healthcare resources [26].

Given the extensive variability in the costs associated with renal complications, the expenses were further categorised into two groups: patients undergoing dialysis and those not on dialysis. For non-dialysis patients, the cost comprised an average of expenditures across different treatment modalities for individuals diagnosed with chronic kidney disease stages I to IV. Conversely, the annual cost for dialysis was determined by a weighted average of the expenses incurred by dialysis patients with end-stage renal disease [27]. The weights were based on the incidences of hemodialysis and continuous ambulatory peritoneal dialysis. The routine HFrEF outpatient care cost, which included clinic visits, relevant procedures, and investigations was also obtained from local published literature [28]. The cost of adverse events was estimated through a straightforward costing approach, adhering to the standard treatment algorithm for urinary tract infections and genital mycotic infections [24].

### Statistical analysis

The primary outcome of the model was the budget impact of Empagliflozin, projected over the five years. This estimation was based on the difference in total costs between the current scenario with GDMT and the proposed new scenario involving GDMT plus a proportion of patients with an add-on of Empagliflozin. The use of GDMT remained constant throughout the timeframe for both scenarios. The total budget was calculated by summing the costs associated with drug acquisition, cardiovascular death, HF hospitalisation, adverse events, outpatient care, and renal outcomes. Additionally, the budget per year was also determined. Costs were reported in both Malaysian Ringgit (MYR) and US Dollars (USD) without year adjustment. The conversion rate was based on the 2023 purchasing power parity (PPP) to consider Malaysia’s economic productivity and standards (1 USD = 1.56 MYR).

To gauge the robustness of the estimations, a one-way-sensitivity analysis was carried out to pinpoint input parameters with the most significant impact on the overall budget. This analysis involved varying one input parameter at a time while keeping the other parameters constant. Parameters of particular interest included the probabilities of clinical events, along with the costs related to drug acquisition, clinical outcomes, and adverse events. The population input parameters and Empagliflozin cost were varied by ±20%, while clinical outcomes were varied by ±10%. The lower ranges for exploration in the latter were because most of the clinical outcomes reported in the literature were consistently closer to the reference value. The cardiovascular death cost ratio was varied between 1.5 and 4.0 to reflect the potential range of terminal care cost.

### Ethical approval

This study was conducted upon approval by the Malaysian Medical Research and Ethics Committee (MREC) (NMRR-ID-22-00340-BY2). Individual patient consent was not applicable as no patient identifying details were collected.

## Results

The model estimated there would be 138,770 eligible HFrEF patients in the first year (Table 2). By Year 5, under the current treatment with GDMT, this annual number treated grew to 421,075, compared to 421,445 in the GDMT plus Empagliflozin scenario. The rapid increment of annual patients was attributed to the high incidences of HF and relatively higher survival of patients over the shorter period of the analysis. On the other hand, the slightly higher number of patients to be treated in the GDMT plus Empagliflozin scenario was because of the effectiveness of Empagliflozin in reducing the cardiovascular death rate. Aside from the reduced cardiovascular deaths, adding Empagliflozin to GDMT also reduced HF hospitalisations (5.59%) and renal outcomes (8.55%). However, adverse events increased by 5.3%, driven by higher rates of urinary tract and genital infections.

**Table 2:** Population estimation and changes in clinical outcomes for GDMT and GDMT + Empagliflozin.

Events (n)	Year 1	Year 2	Year 3	Year 4	Year 5	Total event	Annual Average	Total difference^1^, (%)	Annual difference^2^, (%)
Population ≥ 18 years	24,935,385	25,184,739	25,436,586	25,690,952	25,947,862	127,195,524	25,439,104		
Incidence of HF	209,457	211,552	213,667	215,804	217,962	1,068,442	213,688		
**Guideline Directed Medical Therapy (GDMT)**									
Cardiovascular death	11,579	17,643	23,588	29,417	35,135	117,361	23,472		
Prevalence of HF	381,824	581,797	777,821	970,037	1,158,582	3,870,061	774,012		
Diagnosed patients	305,459	465,437	622,257	776,030	926,866	3,096,049	619,209		
Treated patients	213,821	325,806	435,580	543,221	648,806	2,167,234	433,446		
HFrEF	138,770	211,448	282,691	352,550	421,075	1,406,535	281,306		
HFrEF with DM	80,487	122,640	163,961	204,479	244,224	815,790	163,158		
Adverse event	5,939	9,049	12,099	15,089	18,022	60,199	12,039		
Cardiovascular death	11,578	17,643	23,587	29,416	35,134	117,361	23,472		
HF hospitalisation	22,184	33,803	45,192	56,360	67,315	224,857	44,971		
Renal composite outcome	4,551	6,934	9,271	11,562	13,809	46,128	9,225		
**Guideline Directed Medical Therapy (GDMT) + Empagliflozin**									
Cardiovascular death	11,494	17,453	23,253	29,007	34,654	115,862	23,172		
Prevalence of HF	381,824	581,881	778,095	970,646	1,159,600	3,872,046	774,409		
Diagnosed patients	305,459	465,505	622,476	776,517	927,680	3,097,637	619,527		
Treated patients	213,821	325,853	435,733	543,562	649,376	2,168,346	433,670		
HF patients with LVEF≤40%	138,770	211,479	282,791	352,771	421,445	1,407,256	281,451		
HF patients with LVEF≤40% and DM	80,487	122,658	164,019	204,607	244,438	816,209	163,241		
Adverse event	6,110	9,441	12,799	15,966	19,074	63,391	12,678	3,191 (5.3%)	638 (1.06%)
Cardiovascular death	11,494	17,453	23,253	29,007	34,654	115,862	23,172	-1498 (-1.3%)	-299 (-0.26%)
HF hospitalisation	21,499	32,241	42,415	52,911	63,211	212,278	42,455	-12,578 (-5.6%)	-2,515 (1.12%)
Renal composite outcome	4,336	6,445	8,400	10,479	12,519	42,183	8,436	-3,945 (-8.6%)	-789 (-1.72%)

^1^ Total difference is defined as the difference between GDMT and GDMT + Empagliflozin over the 5 years.

^2^ Annual difference is defined as the average of the total difference between GDMT and GDMT + Empagliflozin over the 5 years.

Table 3 outlines the financial impact of adding Empagliflozin to a fraction of current patients on GDMT. On average, GDMT alone costs MYR 1.23 billion (USD 78.85 million) annually. With the addition of Empagliflozin, the average annual cost increases by MYR 8.65 million (USD 5.54 million), corresponding to an additional 5-year budget of MYR 43.23 million (USD 27.71 million). The net increment of 0.71% was mainly due to higher drug acquisition costs (an annual increment of MYR 32.02 million; USD 20.53 million) and a slight increase in adverse event costs (MYR 42,308; USD 27,120) and managing additional patients at outpatient care (MYR 167,018; USD 107,062). Despite these large increases, the improvement in clinical outcomes was able to offset most of the expenditures. The average annual savings seen was MYR 14.77 million (USD 9.47 million) from HF hospitalisation, MYR 4.67 million (USD2.99 million) from renal outcomes, and MYR 4.15 million (USD2.66 million) from cardiovascular deaths.

**Table 3:** Expenditures and changes in budget for GDMT and GDMT + Empagliflozin (MYR).

Events	Year 1	Year 2	Year 3	Year 4	Year 5	Total	Annual Average	Total difference, (%)^1^	Annual difference, (%)^2^
**Guideline Directed Medical Therapy (GDMT)**									
Drug acquisition	124,889,758	190,298,341	254,415,341	317,286,843	378,957,608	1,265,847,893	253,169,578		
Adverse event	587,326	894,926	1,196,453	1,492,123	1,782,145	5,952,975	1,190,595		
Cardiovascular death	160,413,499	244,426,952	326,781,442	407,536,164	486,748,609	1,625,906,669	325,181,333		
HF hospitalisation	130,229,905	198,435,287	265,293,858	330,853,675	395,161,413	1,319,974,139	263,994,827		
Outpatient care	160,629,128	244,755,512	327,220,703	408,083,976	487,402,899	1,628,092,220	325,618,444		
Renal composite outcome	26,916,937	41,014,159	54,833,013	68,383,430	81,675,057	272,822,597	54,564,519		
**Total**	**603,666,554**	**919,825,181**	**1,229,740,812**	**1,533,636,213**	**1,831,727,733**	**6,118,596,495**	**1,233,719,299**	** **	** **
**Guideline Directed Medical Therapy (GDMT) + Empagliflozin**									
Drug acquisition	133,500,440	210,009,258	289,599,299	361,264,890	431,591,987	1,425,965,876	285,193,175	160,117,983 (12.65%)	32,023,596 (2.53%)
Adverse event	598,583	920,790	1,242,757	1,550,296	1,852,091	6,164,519	1,232,9903	211,543 (3.55%)	42,308 (0.71%)
Cardiovascular death	159,247,502	241,796,928	322,144,334	401,863,672	480,094,095	1,605,146,533	321,029,306	-20,760,135 (-1.28%)	-4,152,027 (-0.26%)
HF hospitalisation	126,206,419	189,266,601	248,988,898	310,604,851	371,070,005	1,246,136,776	249,227,355	-73,837,362 (-5.59%)	-14,767,472 (-1.12%)
Outpatient care	160,629,128	244,790,919	327,335,974	408,340,058	487,831,233	1,628,927,314	325,785,462	835,093 (0.05%)	167,018 (0.01%)
Renal composite outcome	25,649,306	38,122,383	49,685,880	61,981,380	74,047,237	249,486,188	49,897,237	-23,336,409 (-8.55%)	-4,667,281 (-1.71%)
**Total Budget**	**605,831,382**	**924,906,882**	**1,238,997,146**	**1,545,605,149**	**1,846,486,649**	**6,161,827,209**	**1,232,365,441**		
**Annual Budget Impact** ^**3**^	**2,164,827**	**5,081,700**	**9,256,334**	**11,968,935**	**14,758,916**	**43,230,713**	**8,646,142**	**0.71%**	**0.14%**

^1^ Total difference is defined as the difference between GDMT and GDMT + Empagliflozin over the 5 years.

^2^ Annual difference is defined as the average of the total difference between GDMT and GDMT + Empagliflozin over the 5 years.

^3^ Annual budget impact describes the total additional cost required each year for GDMT + Empagliflozin when compared with GDMT.

**Fig 2:**
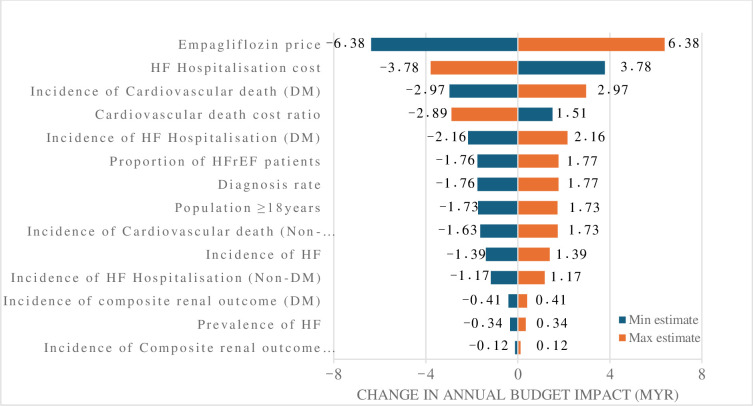
Tornado diagram showing the one-way sensitivity analysis of the budget impact. HF: heart failure, DM: diabetes mellitus, LVEF: left ventricular ejection fraction.

The one-way sensitivity analysis identified the key determinants influencing the budget impact as the cost of Empagliflozin, the expenses associated with HF hospitalizations, and the incidence of cardiovascular mortality in patients with DM mellitus (Fig 2). A 20% reduction in drug price resulted in an annual net budget decrease of MYR 2.27 million (USD 1.45 million), while a 20% price increase raised the net budget by MYR 15.02 million (USD 9.63 million). In contrast, factors such as incidences of renal outcomes, and HF prevalence and incidence, had a minimal effect on budget variation between the two scenarios. A notable finding from Fig 2 was the inverse relationship between HF hospitalisation costs and the budget impact. Reducing HF hospitalisations and cardiovascular death costs ratio (which was also proxied against the HF hospitalisation expenses) led to a higher incremental budget impact, and vice versa.

## Discussion

### Affordability of Empagliflozin

Focusing on the affordability of incorporating Empagliflozin into GDMT for the treatment of HFrEF, the model illustrated that even at a conservative market share, the addition would require a net annual investment of approximately MYR 8.65 million. Overall, the incremental cost of adding Empagliflozin into GDMT of MYR 32.23 million, was predominantly compensated by cost savings from reductions in cardiovascular deaths, HF hospitalisations, and renal composite outcomes of MYR 23.59 million. Reduction in hospitalisations contributed to 62.6% of the cost savings, which corresponded to 2,515 hospitalisations avoided.

Our modest budget increase of 0.71% was shown to be slightly higher than the findings by Karlsdotter et al., in Sweden, which showed an increment of only 0.04% among HFrEF patients [29]. In their study, they applied a similar market share proportion and implementational strategy. The lower proportions in Sweden may be attributed to the higher inpatient costs prevalent in developed countries, such as Sweden. The elevated procedural and resource costs could have diminished the overall impact of medication expenditures on the total budget. In a study by de Beer et al. among patients with DM and established cardiovascular disease in South Africa, a market share ranging from 6% to 10.6% resulted in a net increase of 13.8% over three years. Notably, the substantial 180% increase in drug acquisition costs was partially counteracted by a 23% reduction in clinical event management costs [30]. This further reiterates the postulation that the comparatively lower inpatient costs in low and middle-income nations may lead to a more significant contribution of drug acquisition costs to the overall budget requirements.

### Financial implications

The results of the budget impact analysis underscore the significant economic challenges posed by HF in Malaysia. Based on the current situation, the Malaysian Ministry of Health is anticipated to allocate approximately MYR 1,230 million annually over the next five years for the management of HFrEF. An economic burden study conducted in Malaysia in 2021 found that the overall cost of addressing HF per year amounted to MYR 766.3 million. Within this total, HFrEF contributed substantially, constituting 61% of the burden at MYR 468.5 million [7]. This discrepancy was mainly attributed to methodological variations in cost estimation. In the investigation by Ong SC et al., an annual average cost of MYR 8,033 was applied to the entire HFrEF patient population for burden estimation. In contrast, our current model forecasted clinical outcomes before applying event-specific costs. This method enables a more realistic portrayal of patients across various treatment levels and identifies the areas of management that exert the greatest strain on the budget [31].

Incorporating the newly proposed Empagliflozin into GDMT would result in an average annual budget for drug acquisition amounting to MYR 285.2 million. This constitutes 0.78% of the 2023 national healthcare allocation of MYR 36,330 million. However, when specifically considering the budget for drugs and supplies, this represents a notable increase of 10.2% of the expenditure, based on the 2019 pre-COVID pandemic drug expenditure of MYR 2,801 million [32]. Expanding the market share of Empagliflozin beyond 20% will further incur additional costs but with a larger reduction in mortality and morbidity. For example, based on ad-hoc analysis, to achieve a market share of 90% of Empagliflozin, a net annual additional budget of MYR43.2 million is required. This investment however will lead to an annual reduction of 1,471 cardiovascular deaths, 12,410 HF hospitalisations, and 3,894 composite renal outcomes.

### Policy recommendations

The budget allocations for public healthcare in Malaysia are demarcated into specific accounts, managed by different fund managers. For instance, budgets for acquiring Empagliflozin fall under the drug supply account, which is overseen by the pharmacy or cardiology unit. Therefore, the cost savings derived from factors beyond drugs and supplies typically are not seen within the account. Consequently, one consideration to ensure the availability of funds is the reallocation of potential savings from other budget holders into the drug and supply accounts. While this approach enhances funding availability and spending efficiency, quantifying the precise amount required to be rewired remains challenging due to the complexity and high demand of the public healthcare sector.

Results from the sensitivity analysis highlighted the price of Empagliflozin as one of the major contributors to the budget impact. Although achieving a substantial price reduction may pose challenges, it is beneficial for both policymakers and pharmaceutical manufacturers to engage in negotiations of drug prices. In exchange for favorable pricing, the implementation of bulk purchasing can be explored to capitalise on lower per-unit costs. This approach is particularly viable within the Malaysian public sector, where one of the procurement mechanisms involves a national concession agreement with a designated supplier, enabling price negotiations by the ministry [33]. The financial commitment from the Ministry of Health offers an incentive for the manufacturer to reduce profit margins as the public healthcare sector provides about 75.5% of inpatient care and 64.3% of ambulatory care to the Malaysian population [34].

Another policy option within financial constraints is to consider initiating Empagliflozin for specific high-risk populations, such as those with DM. This rationale stems from the observation that patients with DM experienced more significant reductions in cardiovascular deaths and hospitalisations, leading to larger cost savings compared to non-diabetic patients. This was evidenced by two budget impact analyses conducted in South Korea and Greece among T2DM patients with high cardiovascular risk. The studies revealed that the additional drug acquisition costs for Empagliflozin were offset by a reduction in clinical events, coupled with additional savings from DM management [35, 36]. This also corresponds to the evidence from the EMPA-REG OUTCOME trial, where Empagliflozin demonstrated a reduction in new insulin use by 60% in insulin-naïve patients and reduced the need for dose increments in existing patients [37].

Facilities can also empower patients to consider self-purchasing or cost-sharing of Empagliflozin in public healthcare facilities to increase treatment access. Although the current public healthcare does not allow financial transactions for drugs and supplies aside from the standard fees listed in the 1951 Fees Act, patients can still purchase recommended items directly from their preferred vendors. This is an option that should be offered to HFrEF patients who may not be included in the market share proportion as it can significantly improve their quality of life and productivity. However, it is crucial to provide clear patient education and establish standardised management procedures for treatment adjustments to mitigate the risks associated with polypharmacy.

### Limitations and recommendations

One pivotal area identified in the study was the importance of having accurate population and cost data to inform the policy. To enhance our estimations, further improvements can be made by stratifying clinical events according to age group and baseline characteristics, a task feasible only with the availability of local real-world data. Additionally, as the policy recommendations show that targeting the DM subgroup may provide a greater inclination for policymakers to endorse the additional budget, exploring a more comprehensive DM budget impact model is worthwhile. Integrating models like the Institute for Health Economics Diabetes Cohort Model (IHE-DCM) and the UK Prospective Diabetes Study Outcome Model (UKPDS-OM) enables the incorporation of other macrovascular risk factors for a more comprehensive costing [38, 39]. Lastly, it’s important to note that our findings likely underestimate the true financial impact of adding Empagliflozin, given that they are based solely on the public health provider perspective.

## Conclusion

The integration of Empagliflozin into the GDMT for HFrEF in Malaysia resulted in a modest increase in the overall budget. While there was an increase in drug acquisition and adverse event costs, this was largely offset by a substantial cost reduction associated with HF hospitalisations, cardiovascular deaths, and composite renal outcomes. The budget impact analysis suggests that a marginal additional cost could significantly improve clinical outcomes for patients. However, it is essential to contextualise budget allocations within the framework of Malaysian public healthcare. To enhance the affordability and accessibility of this treatment approach, policymakers can explore price negotiations, and leveraging the demand within the public health sector. Moreover, promoting patient cost-sharing can be instrumental in expanding access to treatment. In situations where budget constraints are a concern, policymakers might contemplate narrowing the target populations to specific high-risk groups.
